# Distribution and Pharmacokinetics of Methamphetamine in the Human Body: Clinical Implications

**DOI:** 10.1371/journal.pone.0015269

**Published:** 2010-12-07

**Authors:** Nora D. Volkow, Joanna S. Fowler, Gene-Jack Wang, Elena Shumay, Frank Telang, Peter K. Thanos, David Alexoff

**Affiliations:** 1 National Institute on Drug Abuse, Bethesda, Maryland, United States of America; 2 National Institute on Alcohol Abuse and Alcoholism, Bethesda, Maryland, United States of America; 3 Brookhaven National Laboratory, Upton, New York, United States of America; Chiba University Center for Forensic Mental Health, Japan

## Abstract

**Background:**

Methamphetamine is one of the most toxic of the drugs of abuse, which may reflect its distribution and accumulation in the body. However no studies have measured methamphetamine's organ distribution in the human body.

**Methods:**

Positron Emission Tomography (PET) was used in conjunction with [^11^C]d-methamphetamine to measure its whole-body distribution and bioavailability as assessed by peak uptake (% Dose/cc), rate of clearance (time to reach 50% peak-clearance) and accumulation (area under the curve) in healthy participants (9 Caucasians and 10 African Americans).

**Results:**

Methamphetamine distributed through most organs. Highest uptake (whole organ) occurred in lungs (22% Dose; weight ∼1246 g), liver (23%; weight ∼1677 g) and intermediate in brain (10%; weight ∼1600 g). Kidneys also showed high uptake (per/cc basis) (7%; weight 305 g). Methamphetamine's clearance was fastest in heart and lungs (7–16 minutes), slowest in brain, liver and stomach (>75 minutes), and intermediate in kidneys, spleen and pancreas (22–50 minutes). Lung accumulation of [^11^C]d-methamphetamine was 30% higher for African Americans than Caucasians (p<0.05) but did not differ in other organs.

**Conclusions:**

The high accumulation of methamphetamine, a potent stimulant drug, in most body organs is likely to contribute to the medical complications associated with methamphetamine abuse. In particular, we speculate that methamphetamine's high pulmonary uptake could render this organ vulnerable to infections (tuberculosis) and pathology (pulmonary hypertension). Our preliminary findings of a higher lung accumulation of methamphetamine in African Americans than Caucasians merits further investigation and questions whether it could contribute to the infrequent use of methamphetamine among African Americans.

## Introduction

Methamphetamine is a highly addictive and toxic drug of abuse. The high addictive potential of methamphetamine is believed to reflect it potency in increasing dopamine in the nucleus accumbens (pharmacological effect associated with drug reinforcement) [1]. Methamphetamine also has potent central and peripheral sympathomimetic effects, which are believed to contribute to its toxic effects [2]. Methamphetamine is relatively easy to manufacture, which has facilitated its availability worldwide and contributed to increases in its abuse. This coupled to its toxicity has resulted in an increase the number of medical complications and fatalities associated with the abuse methamphetamine [3], [4].

Methamphetamine's medical complications affect multiple organs [5]. In brain these include cerebral stroke, hemorrhage, psychoses, seizures; in heart these include myocardial infarction, arrhythmias, cardiomyopathy and ventricular hypertrophy; in lung these include pulmonary edema and hypertension; and in kidneys it includes acute renal failure [6]. Postmortem studies also report pathological findings in the livers of methamphetamine absuers [7]; though this could reflect the high prevalence of hepatitis C infection in this population rather than direct effects of the drug in tissue. Nonetheless the widespread organ toxicity reported in methamphetamine abusers suggests that methamphetamine distributes and is taken up by most organs of the human body as we recently showed to be the case for non-human primates [8].

Here we evaluate the pharmacokinetics and distribution of methamphetamine in the various organs of the human body. Since the abuse of methamphetamine is very low among African Americans (AA) when compared to Caucasians (C) [9], [10] we also compared the distribution of methamphetamine in the body between AA and Caucasians C. For this purpose we used PET and [^11^C]d-methamphetamine to measure the whole body distribution and pharmacokinetics of methamphetamine in vital organs of the human body in healthy non drug abusing males (10 AA and 9 C).

## Results

**Figure 1:**
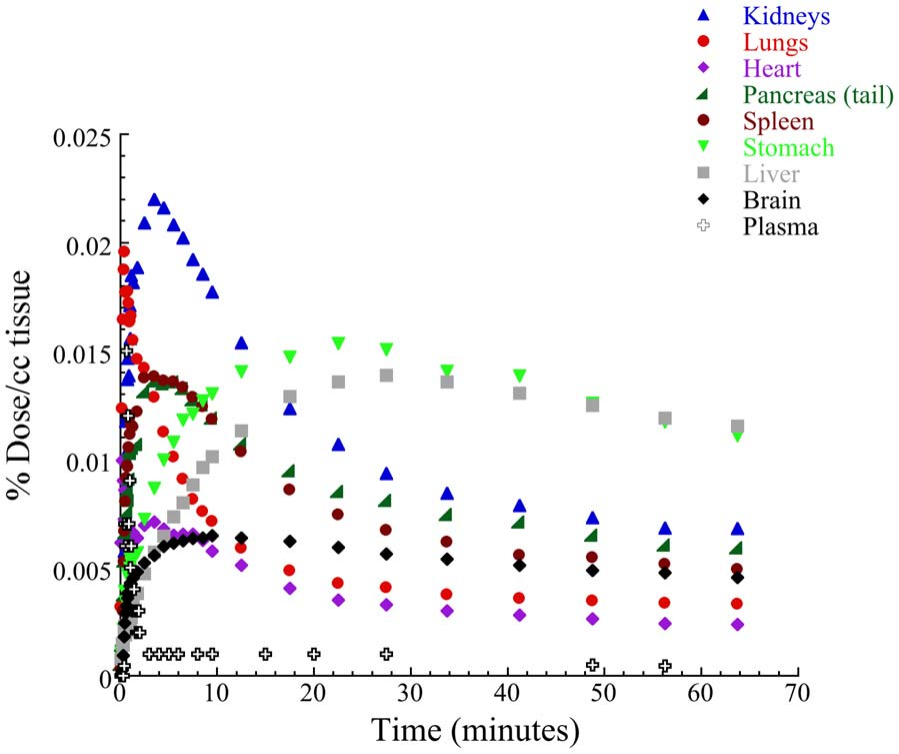
Averaged time-activity curves of [^11^C]d-metamphetamine in the various organs of the body. Note that the values correspond to the average for all subjects (AA and C) whereas Table 2 reports separate values for AA and C, which is why they don't exactly match.

[^11^C]d-Methamphetamine concentration in arterial blood peaked at 50 seconds (0.005±0.004 %Dose/cc) and half peak clearance occurred at 90 seconds (Figure 1, Table 1). Percent excretion of radiotracer in urine was 6.5±2.4 at 87±4 min post [^11^C]d-methamphetamine injection and did not differ between AA (6.9±2.7%) and C (5.9±2.2%).

**Table 1:** Uptake and pharmacokinetics of [^11^C]methamphetamine in the various organs.

Organ	Peak time	Half-peak clearance	Peak% Dose/cc	*Organ weight*% Dose organ	AUC
**Plasma** a **AA** **C**	50 sec	1.5 min	0.014±0.0020.014±0.003	*4500 grams* a63%63%	0.029±0.0040.030±0.003
**Lung** **AA** **C**	55 sec	7 min	0.025±0.005*0.019±0.007	*1246 grams* b31.2%23.7%	0.393±0.069*0.294±0.082
**Heart** **AA** **C**	1 min	16 min	0.007±0.0030.007±0.001	*365 grams*2.55%2.55%	0.259±0.0720.205±0.087
**Kidneys** **AA** **C**	3 min	22 mi	0.022±0.0040.022±0.005	*305 grams* b6.7%6.7%	0.732±0.1250.720±0.167
**Pancreas (tail)** **AA** **C**	5 min	50 min	0.013±0.0020.015±0.002	*144 grams*1.9%2.2%	0.518±0.2250.655±0.239
**Spleen** **AA** **C**	3.5 min	30 min	0.014±0.0020.013±0.002	*156 grams*2.2%2.0%	0.513±0.1010.503±0.064
**Liver** **AA** **C**	30 min	>75 min	0.014±0.0030.013±0.002	*1677 grams*23.5%21.8%	0.871±0.2150.778±0.138
**Stomach** **AA** **C**	30 min	>75 min	0.014±0.0070.017±0.005	*330 grams*4.6%5.6%	0.855±0.2150.967±0.457
**Brain** **AA** **C**	9 min	>75 min	0.006±0.0010.006±0.001	*1600 grams*9.6%9.6%	0.371±0.0430.380±0.041

Measures correspond to: time to peak concentration (Peak time), time to half-peak clearance averaged across both groups and peak concentration (expressed as % dose per cc) and AUC for the time activity curves for the African Americans (AA) and for the Caucasians (C).

*Unpaired Student t test (two tail) p<0.05.

**^a^**The plasma value was extrapolated to whole blood assuming a 55% plasma volume.

**^b^**Reflects the total weight of both left and right organs. Note that the total percent of organ accumulation is greater than 100%; this is because the times at which the peak uptake and the clearance occurs differs among the organs. The weight of the organs corresponds to the average weights recorded from male autopsies [37]; except in brain, which corresponds to weights obtained with MRI^38^.

[^11^C]d-Methamphetamine distributed throughout the whole body (Figure 1). The highest peak concentration (% Dose/cc) occurred in kidneys and lungs followed by stomach, pancreas, spleen, liver and lower values in heart and brain (Table 1). The pharmacokinetics of [^11^C]d-methamphetamine also differed between organs; uptake was fastest in lung and heart (55–60 seconds), followed by spleen, kidneys and pancreas (3–5 minutes) and brain (9 minutes) and was slowest in stomach and liver (30 minutes); clearance (half-peak clearance) was fastest for lungs (7 minutes), intermediate for heart (16 minutes), kidneys (22 minutes), spleen (30 minutes) and pancreas (50 minutes) and slowest for brain, stomach and liver (>75 min) (Table 2).

**Table 2:** Number of subjects in whom [^11^C]d-methamphetamine measures were obtained for the various organs.

Organ	African Americans (n = 10)	Caucasians (n = 9)
Heart	8	7
Lungs	8	7
Kidneys	9	6
Stomach	9	7
Liver	10	7
Spleen	10	7
Pancreas	8	5
Brain	10	9

**Figure 2:**
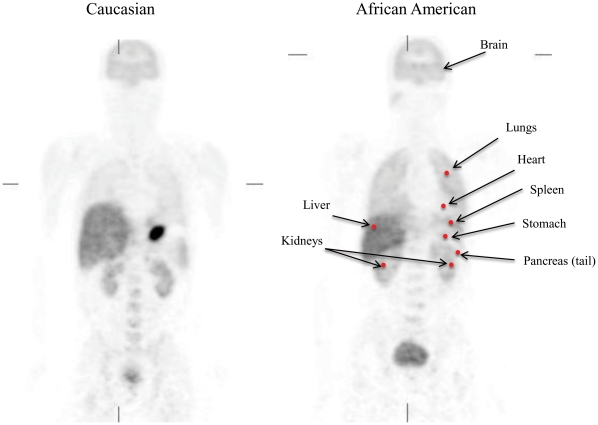
Whole body images of [^11^C]d-methamphetamine in an African American (AA) and in a Caucasian (C) who received 7.18 and 6.99 mCi respectively and location of areas where ROI were obtained. Imaging was started 4 min post injection moving from head to pelvis in 12 minute segments. The images have been decay corrected. Note the higher accumulation of [^11^C]d-methamphetamine in the lung of the AA than of the C. The hot spot on the abdominal cavity of the Caucasian corresponds to the stomach where [^11^C]d-methamphetamine accumulation was high but quite variable across subjects (may reflect its acidic environment that favors trapping of methamphetamine, which is a weak base).

The comparisons between AA and C showed no differences in plasma concentration of radiotracer (Table 1), nor in the percentage of unmetabolized radiotracer in plasma, which at 60 minutes corresponded in AA to 72% and in C to 71%. Lung uptake of [^11^C]d-methamphetamine (expressed as % dose per cc/tissue) was higher in AA (0.025±0.005) than in C (0.019±0.007) (p<0.05) but did not differ in other organs (Figure 2, Table 1). The lung accumulation of [^11^C]d-methamphetamine (AUC) was 33% higher in AA than C (p<0.5) but did not differ in other organs (Table 2).

## Discussion

Here we show that [^11^C]d-methamphetamine is widely distributed in the human body and higher for some organs than for brain (per/cc tissue). The widespread distribution of methamphetamine in the various organs of the body is consistent with our prior findings in non human primates and rats in whom we showed a similar organ distribution of [^11^C]d-methamphetamine [8], [11] and with immunocytochemistry studies in mice [12]. In humans we show that the uptake of [^11^C]d-methamphetamine was highest (per/cc tissue) in kidneys and lungs, intermediate in stomach, pancreas, liver, spleen and lower in brain and heart (Figure 1). This distribution differs markedly from that of cocaine for which we showed that the highest uptake occurred in brain and the lowest in lungs [13].

The lung, along with the kidneys, had the highest [^11^C]d-methamphetamine uptake and at peak approximately 24–31% of the injected dose was in the lung (assuming a weight of 1246 grams). The uptake of [^11^C]d-methamphetamine in lung was very fast (peak 55 seconds) and is unlikely to just reflect its vascular perfusion since the peak concentration in lung was much higher than in arterial plasma and its clearance much slower (7 minutes vs 1.5 minutes). Lung accumulation could reflect uptake by monoamine transporters since [^11^C]d-methamphetamine is a substrate for the dopamine, serotonin and norepinephrine transporters [14] and/or non-specific binding. Of particular clinical relevance would be its uptake by the serotonin transporter since it is the one implicated in the higher risk for pulmonary hypertension seen in methamphetamine abusers [15]. However, to our knowledge there is currently no data showing that the serotonin transporter is responsible for the uptake of methamphetamine in lungs.

A recent report revealed a greater risk of tuberculosis (TB) infection among methamphetamine users than non-users [16]. Similarly a study of HIV-infected patients in Thailand reported that 40% of those also infected with TB had a history of methamphetamine use [17]. Though this probably reflects improper nutrition and/or compromised immune function in methamphetamine abusers, we speculate that the high accumulation of methamphetamine in the lungs may also contribute by rendering pulmonary tissue more vulnerable to infections (or other insults). Studies to evaluate the association between methamphetamine abuse and TB merit more careful evaluation.

The high accumulation of methamphetamine in kidneys (at peak 7% of the injected dose was in the kidneys, assuming 305 grams for both kidneys) could reflect its high urine excretion, which is likely to reflect both its active secretion by renal tubule cells as well as its partition into an acidic urine (methamphetamine is a weak base) [18], [19]. It is estimated that 37–45% of an intravenous or smoked dose of methamphetamine is excreted in the urine as the parent drug and 6–7% as amphetamine within 72 hours of dosing (most of the excretion occurring within the first 20 hours) [18]. This is consistent with our urine measurements which showed 10% of the injected dose was present in urine 90 minutes after injection of [^11^C]d-methamphetamine.

In the pancreas (tail) the high uptake (per/cc tissue) of methamphetamine could reflect uptake by dopamine transporters and vesicular monoamine transporters, which are targets of methamphetamine and are highly expressed in pancreatic tissue [20] and may mediate the rapid increases in insulin that follow acute methamphetamine administration [21]. Though to our knowledge there are no published studies of methamphetamine abuse and diabetes, a preliminary report of abnormal glucose tolerance tests and aberrant insulin values in methamphetamine abusers supports this possibility [22].

Methamphetamine's accumulation in spleen was consistent with similar findings in rats and in non-human primates [8], [11]. In rodents methamphetamine impairs splenic lymphocyte function and produces immunosuppression [23], [24], which could contribute to the higher rate of infections in methamphetamine abusers (i.e., TB, HIV). However, this interpretation requires demonstration of a causal relationship between local concentrations of methamphetamine and immunosuppression, which to our knowledge is currently not available.

The heart had lower methamphetamine uptake than other organs (2.6% injected dose at one min after injection) and its retention in heart was very short lasting. This was unexpected since cardiovascular events are among the most frequent medical complications reported in methamphetamine abusers (review [6]). Thus our findings are consistent with the belief that methamphetamine's central and peripheral sympathomimetic effects rather than direct effects to myocytes, are responsible for its cardiotoxic effects (review [25]). Nonetheless, the good temporal correspondence between methamphetamine's fast accumulation in heart (peaks 60 seconds) and the fast increases in blood pressure induced by this drug (peaks at 60 seconds after iv administration), [26] suggests that methamphetamine may also directly affect cardiac tissue.

Brain uptake of methamphetamine was lower than in many of the organs (per cc of tissue), which could contribute to its clinical toxicity since significant organ accumulation will occur when the drug is used for recreational purposes. This is distinct from cocaine for which the brain uptake is higher than that observed in other organs [13]. On the other hand methamphetamine's clearance from brain was very slow, which is likely to result in long lasting exposure of the brain to the sympathomimetic effects of this drug and contribute to its neurotoxicity. The neurotoxic effects of methamphetamine have been extensively documented and are believed to reflect both its vasoactive effects, which can result in ischemia and necrosis as well as its cathecholaminergic effects, which can result in damage to dopamine neurons, psychosis and seizures [27], [28].

The pharmacokinetics of [^11^C]d-methamphetamine in the stomach and liver were similar and were the slowest from all the organs. The hepatic accumulation of [^11^C]d-methamphetamine was very high (22–24% injected dose; weight 1677 grams) and presumably represents methamphetamine and its metabolites. Some of the liver accumulation could reflect its uptake and excretion through the gallbladder [29]. The unexpected high accumulation in stomach is likely to reflect the acid environment that favors the uptake of a basic drug such as methamphetamine. We note that the accumulation in stomach was quite variable among subjects, which could reflect in part differences in stomach acidification [30].

[^11^C]d-Methamphetamine's uptake and accumulation in lung was higher in AA than C. This is noteworthy since the prevalence rates of methamphetamine use in AA are much lower than in C [9], [10]. Cultural factors as well as market factors in drug access are likely to contribute to these differences. However, differences could also reflect genetic factors that make AA more vulnerable to the adverse effects of methamphetamine. Indeed the most consistent and robust findings in the genetics of drug abuse are that of “protective” genes that confer an enhanced sensitivity to the untoward effects of the drug (i.e., ALDH gene that leads to impaired metabolism of alcohol and protection against alcoholism) [31]. Similarly in rats an enhanced sensitivity to the aversive effects of methamphetamine was associated with lower rates of drug self-administration. Interestingly, the serotonin transporter gene was implicated in this association [32].

The following are study limitations. First, PET measures the concentration of carbon-11 and cannot ascertain if the measures correspond to the drug or labeled metabolites. However, analysis of the plasma revealed that 70–72% of the parent compound remained at 60 minutes after injection indicating that the bulk of carbon-11 in most organs corresponded to the parent compound. Second, these studies were done at tracer doses of [^11^C]d-methamphetamine, which raises the question of whether the pharmacokinetics of a tracer dose reflects that of a pharmacologically active dose (i.e., 0.5 mg/kg). However, the fact that the pharmacokinetics of d-methamphetamine in the baboon brain were not altered at behaviorally active doses [8] suggests that tracer doses reflect those of pharmacological doses. Third, from these studies we cannot determine whether methamphetamine's uptake (methamphetamine is a substrate for monoamine transporters) is by specific reuptake sites or non-specific accumulation. Fourth, methamphetamine is a mixture of levorotatory and dextrorotatory isomers and here we report only on d-methamphetamine. Though this is the predominant form of methamphetamine sold in the streets some forms contain l-methamphetamine and thus one could question whether this could result in a different organ distribution from that of d-methamphetamine. This is unlikely since we had previously shown no differences in the uptake and distribution of carbon-11 labeled l- methamphetamine and d- methamphetamine in non-human primates [33]. Finally, the sample size was small and thus we are treating the differences in pulmonary accumulation of [^11^C]d-methamphetamine between AA and C as preliminary and in need of replication.

In summary this study reports widespread distribution of methamphetamine throughout most body organs, which is likely to contribute to the serious medical conditions that affect methamphetamine users. It also identifies differences in pulmonary uptake of methamphetamine between AA and C that merits further investigation.

## Materials and Methods

### Studies in Humans

#### Subjects

Ninteen healthy male controls (9 C and 10 AA), matched for age (C 39±7; AA 36±5) were recruited by word of mouth and newspaper advertisements. A complete physical and neuropsychiatric examination was done to exclude physical illnesses or neuropsychiatric disorders including substance use disorders (illicit drugs, alcohol or nicotine). Care was taken to ensure that subjects were not smokers, to avoid potential confounds secondary to radiotracer retention in lungs [34]. Subjects were also excluded if they were currently (past 2 weeks) on prescription or over the counter medications. A qualitative immunoassay in urine was done prior to each PET scan to ensure that no psychoactive drugs had been used. This study was carried out at Brookhaven National Laboratory and approved by the local Institutional Review Board (IRB of record: Committee on Research Involving Human Subjects (CORIHS); Study #: IRBnet #91581; CORIHS ID #2007-4835; BNL IRB #373) and written informed consent was obtained from all participants.

Seventeen of the subjects underwent a torso dynamic scan and for twelve of them, we were able to visualize both heart and kidneys, but for five subjects we had to perform a second dynamic torso scan to image thoracic and abdominal organs. We could not visualize all of the organs on all of the subjects. Table 2 provides the sample size for each organ for each of the races. Three of the subjects underwent a segmented whole body scan. The subjects also had two brain PET scans (with [^11^C]d-methamphetamine and with [^11^C]cocaine, which were reported previously in a study that compared the brain uptake of [^11^C]d-methamphetamine and of [^11^C]cocaine [35]).

#### Radiotracer Synthesis

[^11^C]d-Methamphetamine was prepared from d-amphetamine and [^11^C]methyl iodide as described previously [8].

#### PET scanning

Dynamic PET imaging was carried out on a Siemen's HR+, whole body PET scanner (4.5×4.5×4.8 mm FWHM at center of field of view) in 3D acquisition mode, 63 planes. For all scans, a transmission scan was obtained with a ^68^Ge/^68^Ga rotating rod source before the emission scan to correct for attenuation before each radiotracer injection. The specific activity of [^11^C]d-methamphetamine was 0.18±0.09 Ci/micromol at time of injection and the dose injected averaged 6.8±0.77 mCi. The radiochemical purity was >98% and the cold compound injected averaged 9.6±6.9 micrograms. For the torso dynamic scans scanning was carried out for 75 minutes with the following time frames (1×10 sec; 12×5 sec; 1×20 sec; 1×30 sec; 8×60 sec; 4×300 sec; 6×450 sec); for the abdomen and the brain dynamic scanning was carried out for 90 minutes. For consistency purposes we only report the data collected up to 70 minutes post injection. For the segmented whole body scans, scanning from brain to pelvis was begun 4 min post [^11^C]d-methamphetamine injection for a total of 7 segments (12 min/segments with 8 minutes emission and 4 minutes transmission). Thus the segment of the image covering the brain is done at an earlier time (5–18 minutes after radiotracer injection) than the segment covering the pelvis (72–80 min after radiotracer injection) so this must be taken into account when viewing the whole body scan. Note that all of the radioactivity measures are decay corrected.

In order to obtain time activity curves in all organs (located in brain, thorax and abdomen) in a given subject we had to do at least two separate studies with [^11^C]d-methamphetamine (one to cover the head and another to cover thorax/abdomen) and some cases required a third study in order to get data in all of the organs. Typically brain scans and torso scans were performed on the same day with a two hour interval between them. When a third scan was necessary to cover all of the peripheral organs, it was performed on a separate day. Arterial blood was sampled automatically (Ole-Dich, Denmark) and assayed for the concentration of parent radiotracer in the arterial plasma [8]. The results for the distribution of [^11^C]d-methamphetamine in brain have been published [35] and here we will only report on these to serve as comparison with other organs.

In ten subjects (6 AA and 4 C) we collected urines at the end of the study (87±4 min post [^11^C]d-methamphetamine injection) to measure the percent of radiotracer excreted in urine.

### Image Analysis

Time frames were added over the experimental period (75–90 min), the planes of the summed images were summed in groups of two for the purpose of region of interest (ROI) placement. In the heart, elliptical ROI were placed directly across the wall of the left ventricle and/or the interventricular septum for three consecutive slices. Circular ROI were placed on each of 3 planes of the left lung, liver, stomach and spleen using the Herbener Atlas as reference [36]. Elliptical regions were drawn on 1–3 planes of the tail of the pancreas (tail was selected to standardized the location of ROI placement) and on 2–5 planes in left and right kidneys. For the brain we averaged the activity in ROI located in cortical, subcortical and cerebellar regions as described previously [35]. Carbon-11 concentration in each ROI was divided by the injected dose and corrected by the subject's weight to obtain the concentrations of C-11 vs. time. We also measured the arterial concentration of the radiotracer and the percentage of non-metabolized radiotracer at different times during the scan. The time activity curves (expressed as % injected dose/cc vs time) for the various organs was obtained and used to measure the time to reach peak concentration, the time to reach half-peak from clearance and the area under the curve (AUC). Differences in these measures between C and AA were tested using unpaired student t tests (two-tail).
